# Severe pityriasis rubra pilaris complicated with Kaposi's varicelliform eruption and cutaneous MRSA infection case report

**DOI:** 10.1016/j.heliyon.2024.e33750

**Published:** 2024-06-27

**Authors:** Gintare Ulianskaite, Fausta Timinskaite, Tadas Raudonis

**Affiliations:** a Vilnius University, Faculty of Medicine, LT-03101, Vilnius, Lithuania; b Institute of Clinical Medicine, Faculty of Medicine, Vilnius University, LT-01513, Vilnius, Lithuania

**Keywords:** Pityriasis rubra pilaris, Kaposi's varicelliform eruption, Herpes simplex virus, Case report

## Abstract

A 62-year-old woman presented to our hospital with erythroderma affecting 100 % of body surface area, skin scaling and a body temperature of 37.3^o^ C. The lesions initially appeared on her scalp 6 months prior, then psoriasis was diagnosed. Topical corticosteroids were prescribed, which were ineffective. After 2 months the rash spread to the rest of the body, accompanied by nail changes and hair loss. The patient was subsequently admitted to the local hospital, where following clinical evaluation, oral methotrexate 10 mg once weekly was initiated for 6 weeks. Despite the administered treatment the patient's health and skin condition deteriorated, manifesting with an appearance of new lesions. By the time of admission to our hospital erythroderma affecting 100 % of body surface area covered with wide skin scales and punched-out erosions on the torso, lower eyelid ectropion, loss of scalp hair and thickened yellow nail plates were observed. Skin biopsy revealed histological changes consistent with pityriasis rubra pilaris diagnosis. Polymerase chain reaction test from erosions confirmed the presence of herpes simplex virus 1/2 and culture results identified methicillin-resistant Staphylococcus aureus. Given the considerations of pityriasis rubra pilaris, hematologic disorders and paraneoplastic syndrome, a comprehensive work-up for haematological and oncological disorders was conducted, which yielded no significant findings. The patient was treated with intravenous corticosteroids, antibiotics, and antiviral drugs. Isotretinoin was initiated following the histological confirmation of pityriasis rubra pilaris. By the time of discharge, the patient's condition improved. During a follow-up visit 43 weeks after the initiation of isotretinoin, the skin was almost clear. The described case highlights the rare possibility of developing Kaposi's varicelliform eruption in patients with pityriasis rubra pilaris and demonstrates that isotretinoin is a safe and effective treatment option for this condition.

## 1 Introduction

Pityriasis rubra pilaris (PRP) is a rare inflammatory papulosquamous disorder of unknown etiology, with the highest incidence occurring in the first and fifth decades of life [1]. Evidence suggests a slight predominance of white individuals, although it stays unclear whether the data is accurate or if the prevalence is elevated due to potential misdiagnosis or underdiagnosis in individuals with darker skin types [[2], [3], [4]]. Traditionally, six types of PRP have been distinguished according to the Griffiths classification. Recently, two new variants have been identified: CARD14-associated papulosquamous eruption (CAPE) and facial discoid dermatosis [4]. Clinically, PRP presents with follicular hyperkeratotic papules that tend to form plaques with characteristic islands of sparing; erythroderma, palmoplantar keratoderma, nail changes, hair sparseness, in severe cases, ectropion [1,4,5]. The pathophysiology remains incompletely understood, however, evidence indicates a key role for dysregulation of the TH17 axis. This is supported by the effectiveness of targeted treatments with IL-17 and IL-23 inhibitors, suggesting significant therapeutic potential [4,[6], [7], [8], [9], [10], [11]]. In previously outlined case reports, the inflammation of PRP as been depicted as being triggered by offending antigens, such as infections, vaccines, ultraviolet exposure, malignancy [1,4,5,11,12]. However, it is important to note that these associations are based on case reports, and causality has not been definitively established [4]. The diagnosis is based on clinical and histological features, including orthokeratosis with alternating spotty parakeratosis, epidermal acanthosis, intact granular layer and mild perivascular lymphohistiocytic infiltrate in the dermis [12]. Furthermore, diagnosis might be challenging in cases of severe erythroderma, which resembles other conditions such as psoriasis, mycosis fungoides/Sezary syndrome, drug eruption, contact dermatitis [4]. The treatment of PRP remains challenging due to the absence of established guidelines. According to the most recent literature, recommended first line therapies include ixekizumab, secukinumab and methotrexate, with high-dose isotretinoin being a potential consideration in cases where immunosuppressive treatment is contraindicated. An adjunctive treatment with emollients, topical corticosteroids or calcineurin inhibitors should also be taken into account [4]. With advancement of understanding the pathophysiology of PRP, biologics have emerged as promising treatment option with a favorable success rate [4,11,13].

We report a severe case of erythrodermic PRP with secondary Kaposi's varicelliform eruption (KVE) and methicillin-resistant Staphylococcus aureus (MRSA) infections successfully treated with isotretinoin.

## 2 Case presentation

**Fig. 1:**
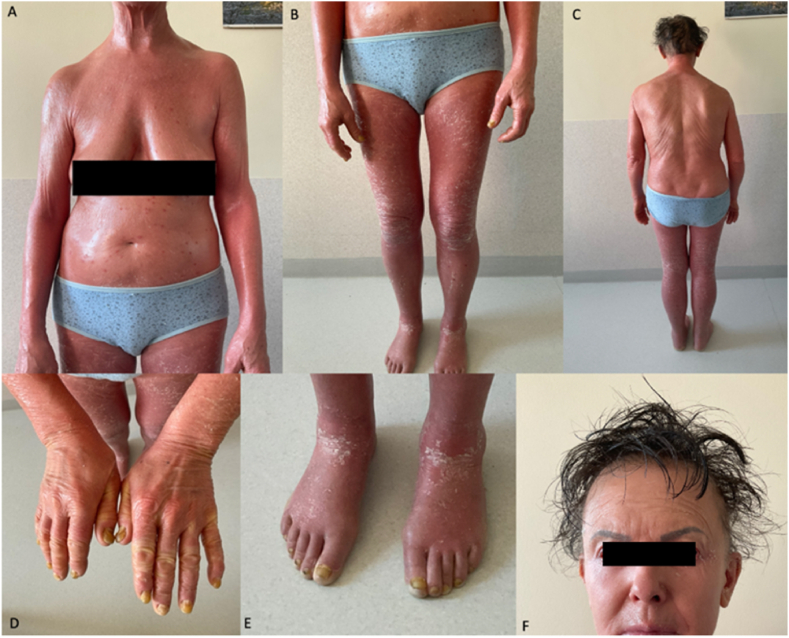
A-C Erythroderma affecting 100 % of body surface area; D-E nail changes, F – ectropion and diffuse hair loss.

**Fig. 2:**
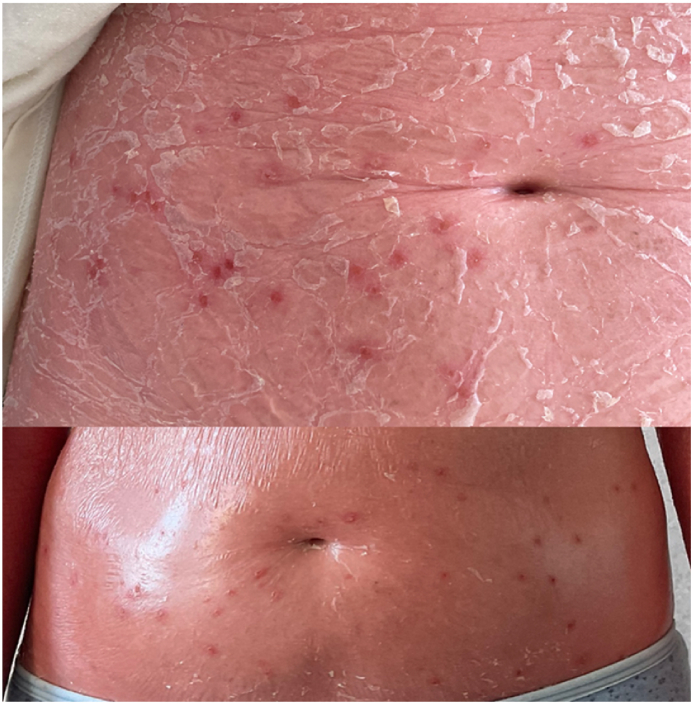
Kaposi's varicelliform eruption on the stomach.

A 62-year-old woman presented to our hospital with erythroderma affecting 100 % of body surface area (BSA), accompanied by skin scaling and a body temperature of 37.3^o^ C (Fig. 1A–F). The initial lesions appeared 6 months prior on her scalp, leading to a diagnosis of psoriasis and the prescription of topical corticosteroids, which were not effective. Two months later, the lesions spread to the rest of the body, accompanied by nail changes and hair loss. Due to the extensive skin involvement, the patient was hospitalized at a local hospital. Biopsy at that time revealed no specific findings. Psoriasis remained the suspected diagnosis, and treatment with oral methotrexate 10 mg weekly was initiated and continued for 6 weeks. Despite immunosuppressive treatment, the patient's health and skin condition dramatically deteriorated with the appearance of new lesions on the torso, thighs (Fig. 2). Additionally, the patient was diagnosed with COVID-19 infection 2 weeks before admission to our hospital. By the time of admission, the patient presented with erythroderma affecting 100 % of BSA, characterised by extensive skin scaling, punched-out erosions on the torso and thighs, lower eyelid ectropion, scalp hair loss and thickened yellow nail plates (Fig. 1 A-F and 2). Movement was painful and complicated due to inflammation. The patient's medical history included depression, nontoxic nodular goiter.

Clinical work-up revealed high-normal leukocyte count (9,85x10^9/l) with mild neutrophilia (7,90x10^9/l), elevated erythrocyte sedimentation rate (ESR) (30 mm/h), and normal C-reactive protein (CRP) (1,3 mg/l). Liver and kidney function tests were within normal reference ranges, despite slight proteinuria (0,2 g/L) and glucosuria (2,8 mmol/L). Additionally, hypoproteinemia (80,2 g/L initially, decreasing to 63,8 g/L after 2 weeks) and secondary cutaneous infections with herpes simplex virus ½, confirmed by polymerase chain reaction test (PCR) and MRSA confirmed by culture from the ulceration were identified. Given the considerations of PRP, hematologic disorders and paraneoplastic syndrome, a comprehensive work-up for hematological and oncological disorders was conducted, with no significant findings noted (Table 1). Two skin biopsies were repeatedly performed. The first biopsy, obtained from erythroderma-affected skin, suggested that histological changes may be compatible with PRP diagnosis. It revealed epidermal parakeratosis, focal hypergranulosis, uneven acanthosis with focal spongiosis; focal extravasation of erythrocytes, single intraepithelial erythrocytes; in the papillary dermis - sparse perivascular lymphocytic infiltration. The second biopsy, taken from a punched-out erosion that appeared 2 weeks before admission, exhibited characteristic changes indicative of a herpes simplex infection with bacterial colonies in necrotic debris, likely representing a secondary infection. Histological examination of this biopsy showed epidermal hyperkeratosis with parakeratosis; area of epidermal detachment with necrotic debris, mixed inflammatory infiltration at the base (including bacterial colonies - in necrotic debris) and scattered multinucleated cells with large nuclei containing intranuclear homogeneous inclusions.

**Table 1:** Detailed clinical work up for PRP differential diagnosis.

Work up	Results
Chest Xray	No infiltrative or focal changes are observed in the lungs
Abdominal ultrasound	Internal organs without sonographic changes
Clonality of TCRB, TCRG, and TCRD genes (DNA fragment analysis by capillary electrophoresis	Non-clonal profile
Peripheral blood Flow cytometry	CD4^+^CD7^−^ and CD4^+^CD26^−^ <250/μL; The Th/Tc lymphocyte ratio remains unchanged. According to flow cytometry, a small amount of Sézary cells present, with a normal CD4:CD8 ratio. Currently, there is no evidence for Mycosis fungoides/Sezary
Microscopy of peripheral blood	No Sezary cells detected
Transvaginal ultrasound	No gynaecological pathology detected
Colonoscopy	No pathology detected
Video Esophagogastroduodenoscopy	No pathology detected

**Fig. 3:**
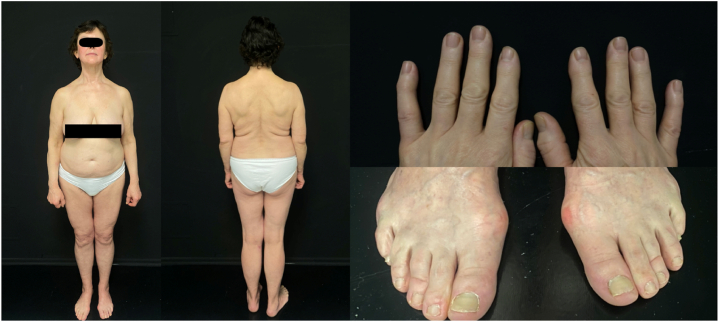
Follow-up visit at 43 weeks of isotretinoin.

The patient received treatment with intravenous corticosteroids, antibiotics (Sulfamethoxazole and Trimethoprim 960 mg twice daily for 10 days), and antiviral drugs (Acyclovir 800 mg x five times daily for 7 days). Isotretinoin 30 mg once daily (0,5 mg/kg) was initiated following histological confirmation of PRP. Upon discharge after 25 days of hospitalization, the patient's condition had significantly improved. During a follow-up visit at 43 weeks of isotretinoin use, the skin was almost clear, nail plates and hair had regrown healthy. Only a few erythematous patches with light scaling were observed on the chest and face (Fig. 3). The patient reported concerns of dry skin and lips due to the treatment, but these symptoms were well controlled.

## 3 Discussion

Severe cases of PRP pose a significant challenge for clinicians in accurately diagnosing the condition and determining the appropriate treatment. Additionally, patients experience a considerable impact on their quality of life [4]. In the case we described, the challenge was further complicated by a concomitant widespread KVE caused by HSV ½, confirmed by PCR method, and a secondary cutaneous bacterial infection with MRSA, while the patient was under immunosuppressive treatment with methotrexate.

KVE is a cutaneous viral infection, which is mainly caused by herpes simplex virus and presents as widespread clustered vesicles that evolve into punched-out erosions [14]. While KVE has been predominantly associated with atopic dermatitis, it can also occur in other skin conditions such as PRP, acantholytic dermatosis, burns, mycosis fungoides and others [14,15]. To our knowledge, to this date there are only 4 case reports of PRP with KVE described in the literature (Table 2). Erdag et al., suggested that KVE in case of PRP could be exacerbated by local corticosteroids and UVB phototherapy [16], while Cavalie et al., provided evidence supporting the potential role of phototherapy in development of KVE [15]. Guenther et al. presented an intriguing case of PRP with KVE and Pseudomonas aeruginosa superinfection, where development of KVE was associated with IL-17A inhibitor use, recent COVID-19 infection, and compromised skin barrier integrity [14]. In our case, the use of immunosuppressive treatment with methotrexate, recent COVID-19 infection and severely damaged skin barrier may have contributed impact to the development of KVE. The patient did not receive phototherapy treatment.

**Table 2:** Detailed summary of Pityriasis rubra pilaris and Kaposi's varicelliform eruption case reports.

Author	Sex	Age	Location	Diagnosis	Treatment	Comments
Ng et al., 1992 [17]	Male	63	Face, neck	Clinical features, culture	I/V aciclovir 5 mg/kg every 8 hours for 3 days, then p/o acyclovir 200 mg 5 times daily for 5 days, ceftriaxone for secondary bacterial infection prevention	First reported case report of PRP complicated by KVE
Erdag et al., 2011 [16]	Male	72	N/I	Histology	P/o Valaciclovir	Associated diminished immunity due to the use of local corticosteroids and UVB phototherapy
Cavalie et al., 2013 [15]	Female	62	Face, trunk	Direct immunofluorescence	P/o Valaciclovir 3 g per day for 10 days	Suggested that phototherapy may have contributed to development of KVE due to reduced cutaneous immunity
Guenther et al., 2023 [14]	Male	60	Axillae, inguinal region, scrotum, legs	Viral culture	P/o valaciclovir 1 g twice daily for 28 days, p/o ciprofloxacin 500 mg twice daily for 10 days	Proposed that use of IL-17 inhibitor and recent COVID-19 infection contributed to the occurrence of HSV infection

CARE checklist: Items 8b, 8d, 12 from CARE checklist are not applicable in this case report, because no diagnostic challenges due to access to testing, financial or cultural reasons were faced, prognosis was not applicable in this case and there is no availability to have patients perspective.

Furthermore, we would like to emphasize the successful treatment outcome achieved with low-dose isotretinoin (0,5 mg/kg). As outlined in the recent review by Tejas et al., consideration of high dose isotretinoin is suggested in cases where immunosuppressive treatment is contraindicated, while low dose isotretinoin might be not effective [4]. In our case, oral retinoids were chosen due to inadequate response to previous immunosuppressive treatment and the absence of reimbursement for biologics for PRP in Lithuania.

However, it is important to acknowledge certain limitations of this case report. This represents the first documented case of PRP with concomitant HSV and MRSA infections, necessitating further investigation to fully elucidate the intricate relationship between PRP and concurrent infections and their causality.

## 4 Conclusion

This case report highlights that clinicians should be aware of the rare potential of developing KVE in PRP patients, necessitating appropriate treatment protocols. Additionally, our patient exhibited a favorable response to low-dose isotretinoin, which, when compared to immunosuppressive therapy, is deemed safer in the context of concomitant infection, and widely accessible.

## Ethics statement

Written informed consent was obtained from the patient for the publication in print and online of all the patient photographs and medical information with the understanding that this information may be publicly available.

## Funding statement

This work received no funding.

## Data availability

The data that support the findings of this case report is available from the corresponding author upon reasonable request.

## CRediT authorship contribution statement

**Gintare Ulianskaite:** Writing – review & editing, Writing – original draft, Visualization, Validation, Supervision, Project administration, Methodology, Investigation, Formal analysis, Data curation, Conceptualization. **Fausta Timinskaite:** Writing – original draft, Visualization, Resources, Methodology, Data curation, Conceptualization. **Tadas Raudonis:** Writing – review & editing, Visualization, Supervision, Resources, Methodology, Formal analysis, Conceptualization.

## Declaration of competing interest

The authors declare that they have no known competing financial interests or personal relationships that could have appeared to influence the work reported in this paper.

## References

[1] Wang D., Chong V.C.-L., Chong W.-S., Oon H.H. (2018). A review on pityriasis rubra pilaris. Am. J. Clin. Dermatol..

[2] Ghatnekar S., Shah N., Min M.S., Mazori D.R., LaChance A.H., Vleugels R.A., Nambudiri V.E. (2022). Clinical features and eosinophilia in pityriasis rubra pilaris: a multicenter cohort. J. Am. Acad. Dermatol..

[3] Halper K., Wright B., Maloney N.J., Kim M.M., Ravi V., Worswick S., Lei D.K. (2020). Characterizing disease features and other medical diagnoses in patients with pityriasis rubra pilaris. JAMA Dermatol.

[4] Joshi T.P., Duvic M. (2024). Pityriasis rubra pilaris: an updated review of clinical presentation, etiopathogenesis, and treatment options. Am. J. Clin. Dermatol..

[5] Klein A., Landthaler M., Karrer S. (2010). Pityriasis rubra pilaris: a review of diagnosis and treatment. Am. J. Clin. Dermatol..

[6] Feldmeyer L., Mylonas A., Demaria O., Mennella A., Yawalkar N., Laffitte E., Hohl D., Gilliet M., Conrad C. (2017). Interleukin 23–helper T cell 17 Axis as a treatment target for pityriasis rubra pilaris. JAMA Dermatol.

[7] Haynes D., Strunck J.L., Topham C.A., Ortega-Loayza A.G., Kent G., Cassidy P.B., Hu R., Choate K., Wang Z., Liu Y. (2020). Evaluation of ixekizumab treatment for patients with pityriasis rubra pilaris: a single-arm trial. JAMA Dermatol.

[8] Haynes D., Reiter T., Velasco R., Chang M., Kulkarni R., Kent G., Strunck J., Cassidy P., Greiling T.M. (2023). Pityriasis rubra pilaris transcriptomics implicate T helper 17 signaling and correlate with response to ixekizumab, with distinct gene expression profiles in nonresponders. J. Invest. Dermatol..

[9] Strunck J.L., Cutler B., Rajpal B., Kent G., Haynes D., Topham C.A., Ortega-Loayza A.G., Yang D., Wang Z., Liu Y. (2022). Pityriasis rubra pilaris response to IL-17a inhibition is associated with IL-17C and CCL20 protein levels. J. Invest. Dermatol..

[10] Boudreaux B.W., Pincelli T.P., Bhullar P.K., Patel M.H., Brumfiel C.M., Li X., Heckman M.G., Pittelkow M.R., Mangold A.R., Sluzevich J.C. (2022). Secukinumab for the treatment of adult-onset pityriasis rubra pilaris: a single-arm clinical trial with transcriptomic analysis. Br. J. Dermatol..

[11] Zhou T., Al Muqrin A., Abu-Hilal M. (2024). Updates on pityriasis rubra pilaris: a scoping review. J. Cutan. Med. Surg..

[12] Roenneberg S., Biedermann T. (2018). Pityriasis rubra pilaris: algorithms for diagnosis and treatment. Acad Dermatol Venereol.

[13] Kromer C., Sabat R., Celis D., Mössner R. (2019). Systemic therapies of pityriasis rubra pilaris: a systematic review. J Deutsche Derma Gesell.

[14] Guenther J.S., Ahronowitz I., Worswick S. (2023). Kaposi's varicelliform eruption after treatment with ixekizumab in a patient with pityriasis rubra pilaris. Cureus.

[15] Cavalié M., Giacchero D., Cardot‐Leccia N., Passeron T., Lacour J.P. (2013). Kaposi's varicelliform eruption in a patient with pityriasis rubra pilaris (pityriasis rubra pilaris herpeticum). Acad Dermatol Venereol.

[16] Erdag G., Lockman D., Tromberg J., Cropley T., Patterson J.W. (2011). A case of pityriasis rubra pilaris with associated focal acantholytic dyskeratosis complicated by Kaposi's varicelliform eruption. J. Cutan. Pathol..

[17] Ng S.K., Ang C.B., Tham A. (1992). Kaposi's varicelliform eruption in a patient with pityriasis rubra pilaris. J. Am. Acad. Dermatol..

